# The fatty acid synthesis gene *fabY* affects lipopolysaccharide synthesis and colistin susceptibility in *Pseudomonas aeruginosa*

**DOI:** 10.1128/spectrum.01339-25

**Published:** 2025-07-15

**Authors:** Dandan Zhou, Xiaoya Wei, Yongxin Jin, Zhihui Cheng, Shouguang Jin, Un-Hwan Ha, Xiaolei Pan, Weihui Wu

**Affiliations:** 1 State Key Laboratory of Medicinal Chemical Biology, Key Laboratory of Molecular Microbiology and Technology of the Ministry of Education, Department of Microbiology, College of Life Sciences, Nankai University 616162, Tianjin, China; 2 Department of Biotechnology and Bioinformatics, Korea University 65692 , Sejong, South Korea; 3 Department of Immunology, School of Basic Medical Sciences, Tianjin Medical University 12610, Tianjin, China; Yan'an University, Yan'an, Shaanxi, China

**Keywords:** *Pseudomonas aeruginosa*, fatty acid, lipopolysaccharide, colistin, *fabY*

## Abstract

Bacterial metabolism impacts susceptibility to antibiotics. Here, we found that mutation of the *Pseudomonas aeruginosa* fatty acid response regulator gene pvrA decreases bacterial susceptibility to colistin. The fatty acid synthesis gene *fabY* is upregulated in the *pvrA* mutant. Deletion of *fabY* in either the *pvrA* mutant or wild-type strain increases bacterial susceptibility to colistin. Further investigation reveals that the mutation of *fabY* enhances lipopolysaccharide production, thereby increasing the surface binding of colistin and bacterial susceptibility.

**IMPORTANCE:**

This project explored the influence of bacterial metabolism on antibiotic sensitivity and found a new mechanism to make *Pseudomonas aeruginosa* sensitive to colistin by interfering with fatty acid synthesis. In this study, it was observed that the mutation of the fatty acid metabolism regulation gene *pvrA* in *P. aeruginosa* reduced the susceptibility of bacteria to colistin. It was also found that the fatty acid synthesis gene *fabY* was up-regulated in the pvrA mutant. Further investigation showed that the *fabY* mutation enhanced the production of lipopolysaccharide and increased the surface binding and bacterial sensitivity of colistin. This study shows that the therapeutic effect can be enhanced by developing fatty acid synthesis inhibitors combined with polymyxin, and it provides a new target for antibiotic treatment.

## OBSERVATION

*Pseudomonas aeruginosa* is an opportunistic pathogen that can cause acute and chronic infections in immunocompromised patients and those with chronic obstructive pulmonary disease, cystic fibrosis, and severe burns (1–6). Due to diverse intrinsic and acquired resistance mechanisms, *P. aeruginosa* exhibits high-level resistance to multiple antibiotics (7–9).

Polymyxins are considered as the last line of defense against extensively drug-resistant gram-negative pathogens. Polymyxins are positively charged cyclic cationic antimicrobial peptides that bind to the lipid A portion of lipopolysaccharide (LPS) via ionic interaction, and then pass through the outer membrane and destroy the inner membrane (10–13). In addition, polymyxins have been shown to inhibit bacterial respiration and promote reactive oxygen species (ROS) production (14).

During lung infections, phosphatidylcholine (PC) serves as a major carbon source for *P. aeruginosa* (15). PC is cleaved by phospholipase C (PlcH), and lipases are secreted by *P. aeruginosa*, resulting in glycerol, choline, and long-chain fatty acids (LCFAs), predominantly palmitic acid and oleic acid (15–17). LCFAs are taken up by bacterial cells and converted into fatty acyl-CoA by fatty acyl-CoA synthetase, and then catabolized through β-oxidation and glyoxylic acid shunt (18, 19). *P. aeruginosa* harbors six fatty acyl-CoA synthetase genes, *fadD1-D6*.

In a previous study using a murine acute pneumonia model, we discovered that the transcriptional regulator PvrA is upregulated during infection and activates genes involved in LCFAs catabolism, including *fadD1* and *fadD6* (20). Additionally, PvrA activates quinolone signaling (PQS) synthesis genes while repressing polyhydroxyalkanoate (PHA) synthesis genes in response to palmitic acid (20, 21). Our results revealed that palmitoylcoenzyme A serves as a ligand of PvrA, which promotes the binding of PvrA to its target DNA (20, 21).

Bacterial metabolism has been shown to influence antibiotic resistance (21, 22). In this study, we investigated the role of PvrA in bacterial susceptibility to antibiotics in response to palmitic acid. We found that the mutation of *pvrA* increases bacterial survival in the presence of colistin. By exploring the PvrA-mediated regulatory pathway, we demonstrated a role of FabY (a key enzyme for *de novo* synthesis of fatty acids) in bacterial susceptibility to colistin. Further studies demonstrated that mutation of *fabY* increases surface LPS levels, which promotes colistin binding. Therefore, our findings reveal a relationship between *de novo* fatty acid synthesis, LPS production, and susceptibility to colistin.

**Fig 1:**
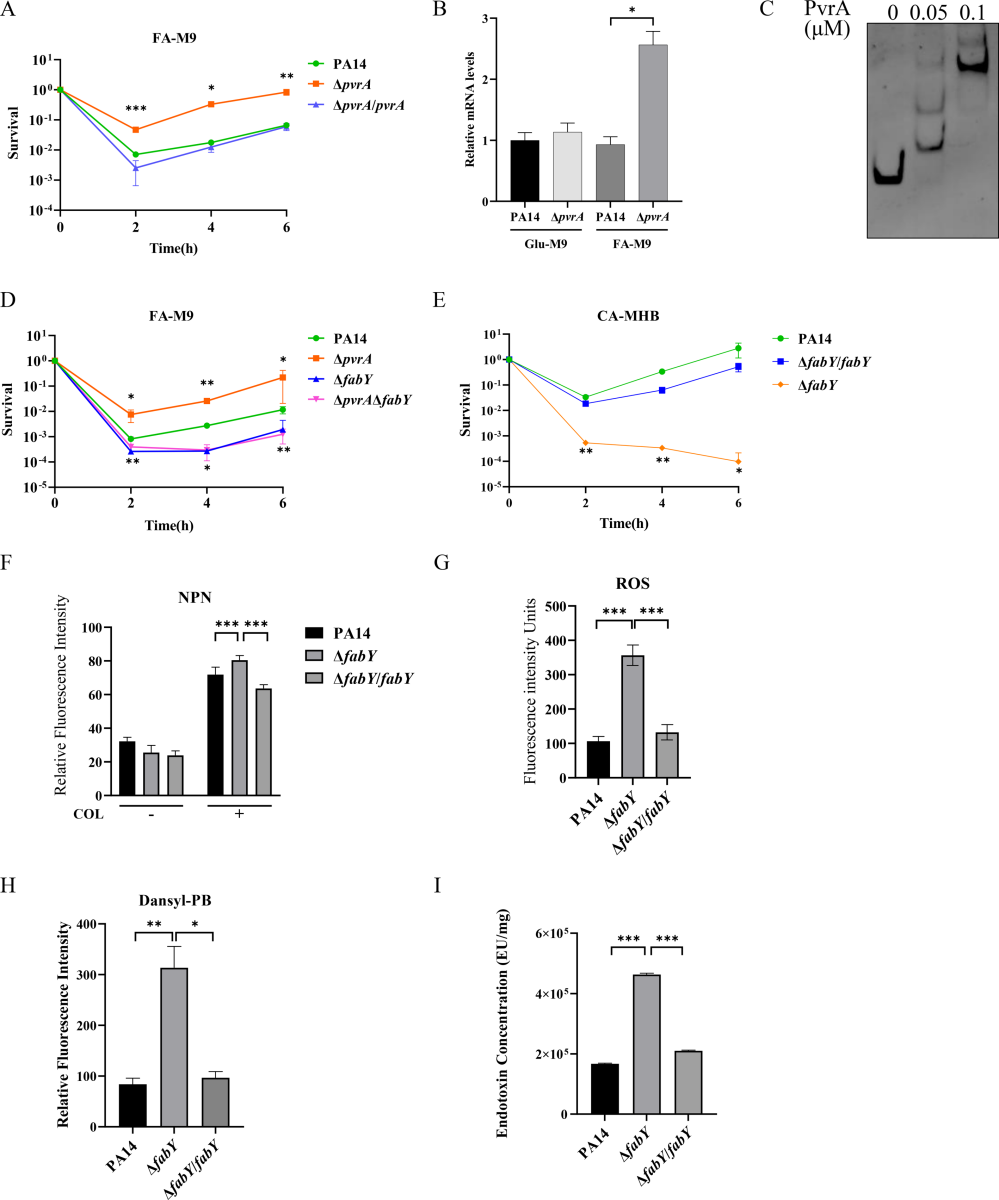
Roles of PvrA and FabY in bacterial susceptibility to colistin. (**A**) Bacteria were grown in the FA-M9 medium until OD_600_ = 1, followed by treatment with 1 µg/mL colistin. Bacterial survivals were determined at the indicated time points. *, *P* < 0.05; **, *P* < 0.01; ***, *P* < 0.001 compared with the wild-type PA14 strain at the corresponding time point by Student’s *t* test. (**B**) Determination of *fabY* mRNA levels in bacteria grown in the FA-M9 medium by RT-qPCR. *, *P* < 0.05 by Student’s *t* test. (**C**) EMSA using PvrA and the promoter region of *fabY*. (**D, E**) Bacteria were grown in the FA-M9 (**D**) or CA-MHB (**E**) medium and treated with 1 µg/mL colistin. Bacterial survivals were determined at the indicated time points. *, *P* < 0.05; **, *P* < 0.01 compared with the wild-type PA14 strain at the corresponding time point by Student’s *t* test. Mutation of *fabY* increases colistin-caused damage and LPS production. (**F**) Bacteria were grown in CA-MHB and treated with 0.5 µg/mL colistin, followed by staining with NPN to determine outer membrane integrity. (**G**) The reactive oxygen species (ROS) levels were determined by incubation of the bacteria with 0.5 µg/mL and 30 µM DCFH-DA at 37°C for 30 minutes. (**H**) The bacteria were incubated with dansyl-polymyxin B at 25°C in the dark for 5 minutes. (**I**) LPS levels were determined by LAL quantification. *, *P* < 0.05; **, *P* < 0.01; ***, *P* < 0.001 by Student’s *t* test. The data represent the mean ± standard deviation of the results of three samples.

To investigate the role of PvrA in bacterial susceptibility to antibiotics, we conducted bacterial killing assays in an M9 minimal medium with palmitic acid as the sole carbon source (FA-M9). The deletion mutation of *pvrA* did not affect bacterial susceptibility to ciprofloxacin or meropenem (Fig. S1). However, the survival of the Δ*pvrA* mutant was higher than the wild-type strain following colistin treatment, which was restored by complementation with a *pvrA* gene (Fig. 1A).

To elucidate the mechanism underlying PvrA-regulated bacterial susceptibility to colistin, we analyzed our previous RNA Sequencing (RNA-Seq) results that compared gene expression profiles between wild-type PA14 and the Δ*pvrA* mutant grown in FA-M9 (23). The results revealed that mutation of *pvrA* increased the expression of the *fabY* (PA14_68360) gene that encodes the β-acetoacetyl-acyl carrier protein synthase, a key enzyme in initiating fatty acid biosynthesis in *P. aeruginosa* (23). Given that disrupting fatty acid synthesis has been shown to enhance the efficacy of colistin against *Escherichia coli* and *Klebsiella pneumoniae* (24), we examined whether FabY is involved in PvrA-regulated bacterial susceptibility to colistin.

RT-qPCR results confirmed the upregulation of *fabY* in the Δ*pvrA* mutant in FA-M9 (Fig. 1B). Electrophoretic mobility shift assay (EMSA) demonstrated the binding of PvrA to the promoter region of *fabY* (Fig. 1C), indicating direct regulation of *fabY* by PvrA. The *fabY* gene encodes the β-acetoacetyl-acyl carrier protein synthase, a key enzyme in the initiation of *de novo* fatty acid biosynthesis (23). A previous study demonstrated that mutation of *fabY* reduced the resistance (MIC) of *P. aeruginosa* to certain antibiotics, including β-lactam, tetracycline, macrolide and aminoglycoside antibiotics, and rifampicin, demonstrating a role of FabY in antibiotic resistance (25). We thus deleted *fabY* in the Δ*pvrA* mutant and wild-type PA14, which reduced the bacterial survival of both strains following colistin treatment in FA-M9 (Fig. 1D). Then, we investigated the role of FabY in a complete medium, cation-adjusted Mueller Hinton broth (CA-MHB). Deletion of *fabY* in wild-type PA14 reduced the bacterial growth rate in CA-MHB (Fig. S4). Meanwhile, the survival of the Δ*fabY* mutant was decreased following colistin (1 × MIC) treatment in CA-MHB (Fig. 1E). These results revealed an important role of FabY in bacterial susceptibility to colistin.

Next, we explored the mechanism of the increased susceptibility of the Δ*fabY* mutant. The bacteria were treated with 0.5 µg/mL colistin for 30 minutes, which did not cause cell death. Compared to wild-type PA14, colistin caused more severe outer membrane damage (Fig. 1F) and ROS production in the Δ*fabY* mutant (Fig. 1G). We then synthesized a dansyl-labeled polymyxin B as previously described (26–28). Deletion of *fabY* increased the binding of the dansyl-labeled polymyxin B to the bacterial surface (Fig. 1H). Since colistin binds to LPS, we quantified LPS using the limulus amebocyte lysate (LAL) assay (29). The Δ*fabY* mutant produced more LPS than wild-type PA14 (Fig. 1I).

**Fig 2:**
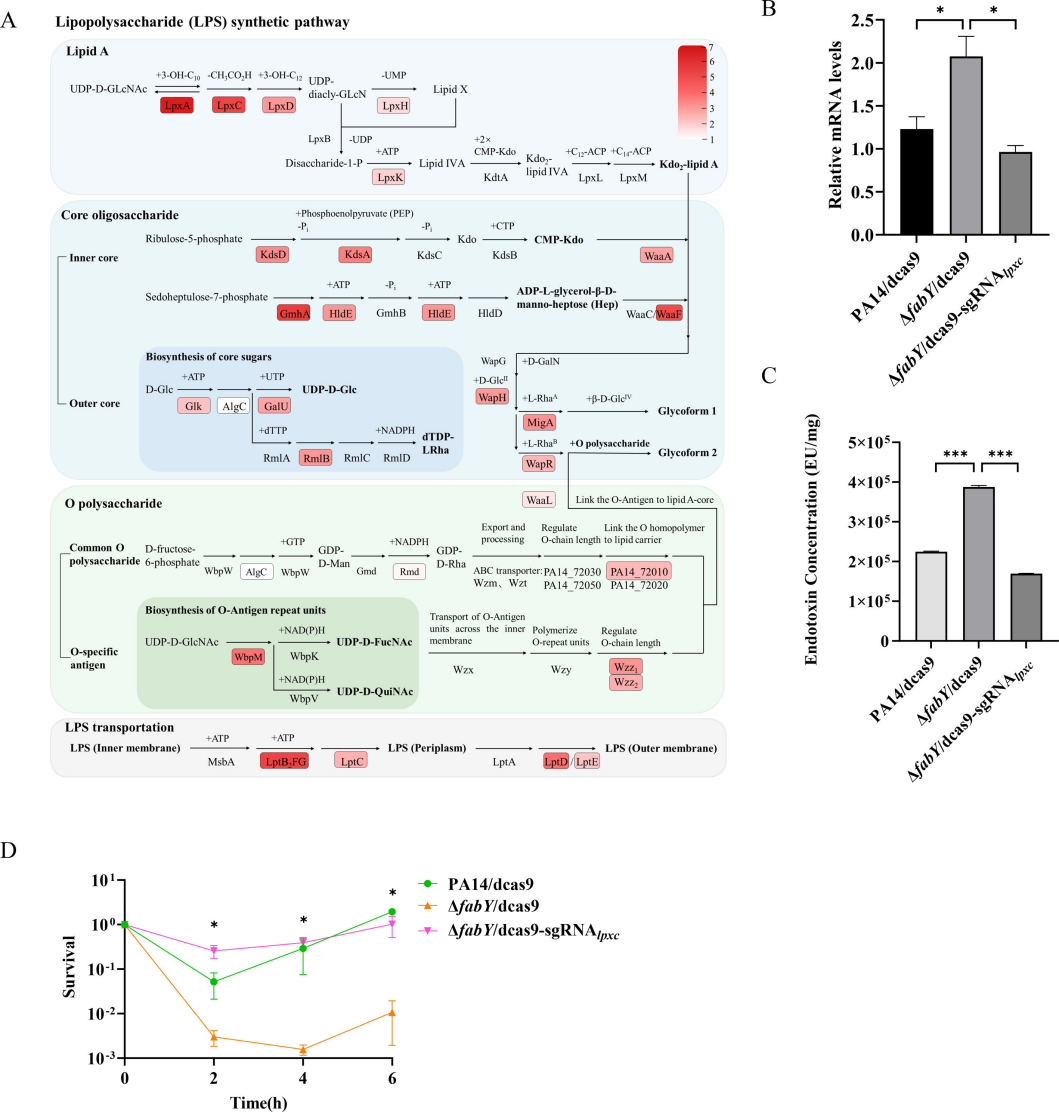
Mutation of *fabY* affects expression of LPS synthesis and transportation genes. Expression levels of the boxed genes were detected by RT-qPCR. The color intensities represent the expression levels in the Δ*fabY* mutant compared to those in wild-type PA14. (**A**) Knock-down of *lpxC* in the Δ*fabY* mutant (Δ*fabY*/dcas9-sgRNA_*lpxC*_) increases bacterial survival following colistin treatment. The expression of dCas9 was induced by IPTG in bacteria with or without *lpxC* targeting gRNA. (**B**) mRNA levels of *lpxC* were determined by RT-qPCR. (**C**) LPS production was determined by the LAL assay. *, *P* < 0.05; ***, *P* < 0.001 by Student’s *t* test. (**D**) Bacterial survival following colistin treatment. *, *P* < 0.05 compared with the Δ*fabY* mutant at the corresponding time point by Student’s *t* test. The data represent the mean ± standard deviation of the results of three samples.

We then assessed the expression levels of genes related to LPS production. In *P. aeruginosa*, 55 genes are involved in LPS synthesis, transportation, and assembly (Fig. 2A; Table S3) (30–33). Quantitative reverse transcription polymerase chain reaction (RT-qPCR) was utilized to determine the expression levels of single genes or the first genes in each operon. Except *algC*, all of the tested genes were upregulated by 1.58- to 6.52-fold (Fig. 2A; Fig S2 and Table S3).

To examine whether the upregulation of those genes contributes to LPS hyperproduction and increased colistin susceptibility, we used CRISPR-interference (CRISPRi) to knock down the expression of *lpxC*, encoding a UDP-3-O-acyl-N-acetylglucosamine deacetylase that catalyzes an irreversible key step in lipid A synthesis (34). Induction of dCas9 and the gRNA reduced *lpxC* mRNA level and LPS production (Fig. 2B and C). Meanwhile, the bacterial survival following colistin treatment increased (Fig. 2D). Collectively, these results indicate that mutation of *fabY* enhances LPS production, which increases colistin binding and subsequent bacterial susceptibility.

A previous study reported that the acylation of lipid A is decreased in a Δ*fabY* mutant. Meanwhile, in most lipid A of the Δ*fabY* mutant, the acyl group is replaced by one or two L-4-aminoarabinose (L-Ara4N) moieties (25). We determined the expression levels of *arnT* and *eptA*, which are involved in the addition of L-Ara4N and phosphoethanolamine (pEtN) to the phosphate groups on lipid A, respectively (35, 36). These modifications usually increase bacterial resistance to polymyxins by reducing the positive charges of LPS (37). The *arnT* gene is regulated by the two-component regulatory systems PhoPQ and PmrAB (38, 39), and the *eptA* gene is regulated by PmrAB and ColRS (40, 41). Both *arnT* and *eptA* were upregulated in the Δ*fabY* mutant (Fig. S3). However, the survival of the Δ*fabY* mutant was lower than wild-type PA14 following colistin treatment. These results indicate that the combined effect of LPS overproduction and upregulation of *arnT* and *eptA* resulted in reduced tolerance to colistin.

Polymyxins are considered as the last line of defense against gram-negative bacterial infections. Identification of genes involved in bacterial resistance to polymyxins might facilitate the development of novel strategies to enhance the treatment efficacies. Here we found that the mutation of *fabY* increases *P. aeruginosa* susceptibility to colistin. Fatty acids are essential for LPS and phospholipids biosynthesis (19, 23, 42, 43). In *E. coli*, phospholipid and lipopolysaccharide synthesis pathways are cross-regulated during outer membrane biogenesis (44). The biotin biosynthesis inhibitor MAC13772, which indirectly inhibits fatty acid synthesis, sensitizes *mcr-1* expressing *E. coli* and *K. pneumoniae* to colistin (24). In addition, inhibitors of fatty acid synthesis enzymes, including cerulenin (inhibiting FabF), Triclosan, and Debio1452-NH_3_ (inhibiting FabI), enhance colistin’s efficacy against *mcr-1* expressing *K. pneumoniae* and colistin-resistant *E. coli* (24). Inhibition of fatty acid synthesis alters phospholipid composition and triggers cell envelope stress, which might enhance the interaction between colistin and the inner membrane and subsequent cell lysis (24). Our results demonstrate that mutation of *fabY* enhances LPS production, which increases surface binding of colistin and bacterial killing. Further studies are warranted to explore the regulatory mechanism of LPS hyperproduction in the Δ*fabY* mutant. We speculated that since fatty acid is also required for the synthesis of phospholipid, the key component of membrane, deletion of *fabY* might cause a stress signal of membrane instability. The signal might trigger the upregulation of LPS synthesis genes, as LPS plays an important role in stabilizing the outer membrane. Previous reports show that in *P. aeruginosa*, PA3286 can directly transfer the β-oxidation intermediate octanoyl-CoA to fatty acid biosynthesis, which enables the growth of the Δ*fabY* mutant (45). Overall, our results reveal a novel mechanism of sensitizing *P. aeruginosa* to colistin by interfering with fatty acid synthesis.

## SUPPLEMENTAL MATERIAL

Supplemental material spectrum.01339-25-s0001.docx Tables S1 to S3; Fig. S1 to S5; Supplemental materials.

ASM does not own the copyrights to Supplemental Material that may be linked to, or accessed through, an article. The authors have granted ASM a non-exclusive, world-wide license to publish the Supplemental Material files. Please contact the corresponding author directly for reuse.
