# Supplementary material for: The fatty acid synthesis gene fabY affects lipopolysaccharide synthesis and colistin susceptibility in Pseudomonas aeruginosa
Source: Microbiol Spectr. 2025 Jul 15;13(8):e01339-25. doi: 10.1128/spectrum.01339-25 (PMC12323585; doi:10.1128/spectrum.01339-25)
Supplement: Supplemental material — Tables S1 to S3; Fig. S1 to S5; Supplemental materials. [file spectrum.01339-25-s0001.docx]

**Supplemental Materials**

**Bacterial strains, plasmids and culture media.**

The bacterial strains, plasmids, and primers used in this study are listed in Table S1. Bacterial cells were cultured in Luria-Bertani (LB) broth (10 g/L tryptone, 5 g/L yeast extract, 5 g/L NaCl, pH 7.4). For solid LB medium, 15 g/L agar was added. Medium with palmitic acid as the sole carbon (FA-M9) source was prepared in the M9 medium (49.4 mM Na_2_HPO_4_, 24.0 mM KH_2_PO_4_, 9 mM NaCl, 19 mM NH_4_Cl, 0.5 mM MgSO_4_), supplemented with 0.2% (w/v) Brij-58 and 0.4% (w/v) palmitic acid [1].

**Bacteria killing assays.**

Overnight cultures of bacteria in LB and FA-M9 media were diluted in corresponding fresh media, and grown to exponential phase (5×10^8^ CFU/mL). The bacteria in LB medium were washed with CA-MHB medium and suspended in 3 mL CA-MHB medium, adjusted to 5×10^7^ CFU/mL. The bacteria in FA-M9 medium were diluted to 5×10^7^ CFU/mL with FA-M9 medium in a total volume of 3 mL. Bacteria were treated with indicated antibiotics at 37 °C with shaking at 200 rpm. Survival of the bacteria was determined at the indicated time points (0, 2, 4, 6 h).

**Electrophoretic mobility shift assay (EMSA).**

The EMSA was performed as previously described with minor modifications [2]. The DNA fragment (400 ng) was incubated with 0, 0.05 or 0.1 μM purified recombinant PvrA protein in 20 μL buffer (10 mM Tris–HCl, pH 7.6, 4% glycerol, 1 mM EDTA, 5 mM CaCl_2_, 100 mM NaCl, 10 mM-β-mercaptoethanol) at 37°C for 10 min. An 8% polyacrylamide gel in 0.5× Tris–borate–EDTA (TBE) buffer (0.044 M Tris base, 0.044 M boric acid, 0.001 M EDTA, pH 8.0) was pre-run for 1 h at 100 V. The samples were then run in the gel on ice at 100 V for 1.2 h. Motility shift was observed by staining in 0.5×TBE containing 0.5 μg/mL ethidium bromide. Bands were visualized with a molecular imager ChemiDoc™ XRS + (Bio-Rad).

**Reverse transcription and quantitative polymerase chain reaction.**

Total bacterial RNA was isolated using the Transzol up plus RNA kit (TransGen, Beijing, China). cDNAs were synthesized with a reverse transcriptase and random primers (TransGen, Beijing, China). Real-time PCR was performed using the PerfectStart Green qPCR SuperMix (TransGen, Beijing, China). The 30S ribosomal protein gene *rpsL* was used as the internal control.

**Reactive oxygen species assay.**

Overnight cultures of bacteria in LB were diluted 1: 100 into the CA-MHB medium and grown until the OD_600_=1. Colistin with a final concentration of 0.5 μg/mL and DCFH-DA with a final concentration of 30 μM were added to 1 mL bacterial culture and incubated at 37 °C for 30 min. The bacteria were then washed three times with PBS and resuspended in PBS. The fluorescence was measured using a microplate reader (Varioskan Flash; Thermo Scientific) at an excitation wavelength of 488 nm and an emission wavelength of 525 nm. All the tests were performed in triplicate.

**Polymyxin B binding assay.**

The synthesis of dansyl-labeled polymyxin B and measurement of the surface bound dansyl-polymyxin B were performed as described previously [3, 4]. 40 mg of polymyxin B sulfate was dissolved in 1.2 mL NaHCO_3_ solution (8.4 mg/mL). 10 mg dansyl chloride was dissolved in 0.8 mL acetone. The two solutions were mixed and reacted at room temperature for 90 min. The product was then loaded to a purification column packed with dextran gel column particles (Sephadex G-25), and eluted with an equilibrium solution (1.42 g/L Na_2_HPO_4_, 8.47 g/L isopropanol, PH=7.0). A total volume of 20 mL eluent was collected.

Overnight bacterial cultures were diluted 1: 100 into LB medium and grown at 37 °C until the OD_600_=1. The bacteria were washed and resuspended with normal saline, and diluted ten fold with normal saline. Then the bacteria were incubated with 5 μg/mL dansyl-polymyxin B at room temperature for 5 min in dark. The samples were washed twice with 0.9% NaCl and resuspended, and then transferred to a black 96-well microtiter plate. The fluorescence was measured using a microplate reader (Varioskan Flash; Thermo Scientific) at an excitation wavelength of 340 nm and an emission wavelength of 485 nm. All the tests were performed in triplicate. The relative fluorescence intensity of the bound dansyl-polymyxin B was calculated as [100×(F-F_0_)/F_0_]%. F, fluorescence intensity of bacteria with dansyl-polymyxin B; F_0_, fluorescence intensity of bacteria without dansyl-polymyxin B.

**Assessment of the outer membrane integrity.**

The integrity of the outer membrane was measured by the fluorescent probe 1-N-phenylnaphthylamine (NPN) as previously described with minor modifications [5]. Overnight bacterial cultures were diluted 1: 100 into CA-MHB medium and grown at 37 °C until the OD_600_=1. The bacterial liquid was adjusted to OD_600_=0.5 with fresh CA-MHB medium, and incubated at 37 °C for 0.5 h without or with polymyxin E at a concentration of 0.5 μg/mL (1×MIC). Next, the bacteria were washed three times with 5 mM GHEPES buffer (Caisson Labs) containing 5 mM glucose and resuspended in the same buffer. The fluorescent probe NPN (Macklin) was added to the bacteria at a final concentration of 10 µM. The fluorescence was measured using a microplate reader (Varioskan Flash; Thermo Scientific) at an excitation wavelength of 350 nm and an emission wavelength of 420 nm. All the tests were performed in triplicate.

**Extraction and quantification of LPS.**

LPS was purified using LPS extraction kit (Solarbio, Beijing, China). The LPS was quantified with a ToxinSensor™ chromogenic LAL endotoxin assay kit (GenScript, Nanjing, China) following the manufacture’s instruction.

**Table. S1** Bacterial strains, plasmids and primers used in this study.

| **Strain/ Plasmid /Primer** | **Description** | **Source (Reference)** |
| --- | --- | --- |
| ***P. aeruginosa*** |  |  |
| PA14 | Wild type strain of *Pseudomonas aeruginosa* | [6] |
| Δ*pvrA* | PA14 deleted of *pvrA* | [7] |
| Δ*pvrA*/*pvrA* | Δ*pvrA* with *pvrA* inserted on chromosome with mini-Tn7T insertion; Gm^r^ | [7] |
| Δ*fabY* | PA14 deleted of *fabY* | This study |
| Δ*fabY*/*fabY* | Δ*fabY* with *fabY* inserted on chromosome with mini-Tn7T insertion; Gm^r^ | This study |
| Δ*fabY*/pHERD20T-dcas9-sgRNA*_lpxC_* | Δ*fabY* knocked down the expression of *lpxC* by CRISPR-interference (CRISPRi); Cb^r^ | This study |
| ***E. coli*** |  |  |
| DH5α | F^-^, φ80d*lacZ*ΔM15, Δ(*lacZYA*-*argF*) U169, *deoR*, *recA1*, *endA1*, *hsdR17*(r_k_^-^, m_k_^+^), *phoA*, *supE44*, λ^-^ , *thi-1*, *gyrA96*, *relA1* | TransGen |
| **Plasmid** |  |  |
| pUC18T-mini-Tn7T-Gm | mini-Tn7 base vector from insertion into chromosome attTn7 site; Gm^r^ | [8] |
| pEX18Tc-mini-Tn7T-Gm-Δ*pvrA* | pUC18T-mini-Tn7T-Tc with *pvrA*; Gm^r^ | This study |
| pEX18Tc-mini-Tn7T-Gm-Δ*fabY* | pUC18T-mini-Tn7T-Tc with *fabY*; Gm^r^ | This study |
| pHERD20T-dcas9 | dcas9 was inserted downstream of arabinose promoter on shuttle plasmid pherdb20; Cb^r^ | [9] |
| **Primer** | **Sequence (5’→3’)** | **Function** |
| *fabY*-UP-F | CCGGAATTCACTCCAGGGAAGAAGCCGAG | *fabY* deletion |
| *fabY*-UP-R | GTTGTGGCGGCGCTCAACCGGTAGTCGAGACATCACTC | *fabY* deletion |
| *fabY*-DOWN-F | GAGTGATGTCTCGACTACCGGTTGAGCGCCGCCACAACG | *fabY* deletion |
| *fabY*-DOWN-R | CGCGGATCCTGGGCGTCGATCGCATC | *fabY* deletion |
| *lpxC-KD-F* | CTAGGAGGTGACTAAGTCGAC TTTGACAGCTAGCTCAGTCCTAGGTATAATGCTAGT  CTTGATGCGGATGAACTTCT GTTTTAGAGCTAG | *lpxc* knockdown |
| *lpxC-KD-R* | GCCAAGCTTGCATGCCTGCAG AAAAAAAGCACCGACTCGG | *lpxc* knockdown |
| *rpsL*-RT-F | GTAAGGTATGCCGTGTACG | RT-qPCR |
| *rpsL*-RT-R | CACTACGCTGTGCTCTTG | RT-qPCR |
| *lpxA*-RT-F | TGACCACAACGTGATCCGC | RT-qPCR |
| *lpxA*-RT-R | ATCACGCTGTCATGGCCG | RT-qPCR |
| *lpxC*-RT-F | AGTCGGTGAACTACCGCGAC | RT-qPCR |
| *lpxC*-RT-R | AGGAACGCCGGGATATCCAG | RT-qPCR |
| *lpxD*-RT-F | AATCCCTACCTGGCCTATGCC | RT-qPCR |
| *lpxD*-RT-R | ACTCTCGATCACCGCATAGGC | RT-qPCR |
| *lpxH*-RT-F | TGTACATCCTCGGCGACTTCTTC | RT-qPCR |
| *lpxH*-RT-R | AGAACGCCTTGCCGATGAG | RT-qPCR |
| *lpxK*-RT-F | ATGATCCTCTGGATGATCGAGCAC | RT-qPCR |
| *lpxK*-RT-R | ACGATCCGGGTCGATCATCAG | RT-qPCR |
| *kdsA*-RT-F | AGGAGATGAAGCACATCCTGACC | RT-qPCR |
| *kdsA* -RT-R | TGGGTGACGTCGAAGAATACCG | RT-qPCR |
| *kdsD*-RT-F | ACATGGGCATGATCACCAAGGAC | RT-qPCR |
| *kdsD*-RT-R | AGGTTCACTTCCGCAGCCTTG | RT-qPCR |
| *gmhA*-RT-F | AACTGCTCAACCGCTTCGAG | RT-qPCR |
| *gmhA*-RT-R | ATGGCCTGGATCACGTTGG | RT-qPCR |
| *hldE*-RT-F | AACGTCGCGCTGAACATCG | RT-qPCR |
| *hldE*-RT-R | TATCGATGCGCTGGAAGCG | RT-qPCR |
| *waaA*-RT-F | AGCTGCTGTTCCTCTATGCC | RT-qPCR |
| *waaA*-RT-R | TCGAGGAAGTTGAACAGGTGCG | RT-qPCR |
| *waaF*-RT-F | TCGAAGAACGACCATCCCGG | RT-qPCR |
| *waaF*-RT-R | AATCGTTGGACACCACCGC | RT-qPCR |
| *glk*-RT-F | TCTATGAAATCAGCTGTGCCCTGG | RT-qPCR |
| *glk*-RT-R | TAATGTAGACGCCACCGAGCG | RT-qPCR |
| *algC*-RT-F | ACATCATCTTCGACGTCAAGTGC | RT-qPCR |
| *algC*-RT-R | TGTAGATGCCATCGTCGAAGC | RT-qPCR |
| *galU*-RT-F | ACGACCTGTGCCTGAACCTC | RT-qPCR |
| *galU*-RT-R | ATCACGCCGTACTTGTTGGTC | RT-qPCR |
| *rmlB*-RT-F | TGGACCTGCAATATGCCGTG | RT-qPCR |
| *rmlB*-RT-R | AGCAACTCGTGGAAGTCGTG | RT-qPCR |
| *migA*-RT-F | TCCACGGTTTCCAGCTCTACC | RT-qPCR |
| *migA*-RT-R | AGCATGATGTCGTCCAGGTC | RT-qPCR |
| *wapH*-RT-F | ACTCGTCTGCCGATCGTTG | RT-qPCR |
| *wapH*-RT-R | TTGTAGAGCGTCTCGATCCGC | RT-qPCR |
| *wapR*-RT-F | AAGTACGGCTTCGACCTCAGG | RT-qPCR |
| *wapR*-RT-R | TTGCGGCAACATGATGTCGTC | RT-qPCR |
| *waaL*-RT-F | AGCGCTTACATGATGCTCAGC | RT-qPCR |
| *waaL*-RT-R | ATGTTGCGTAGGCGTTCGG | RT-qPCR |
| *rmd-*RT-F | TGCAGATCAACCTCCTTGGCAC | RT-qPCR |
| *rmd*-RT-R | ATGAGTTCCTCGTGGATCGGC | RT-qPCR |
| *PA14_72010-*RT-F | ATCACCCTCTCCGAACAGACTG | RT-qPCR |
| *PA14_72010*-RT-R | TTGAGTTGCAGGGTCTCGG | RT-qPCR |
| *wbpM-*RT-F | AACTACTGGTGGTTGAGCATGC | RT-qPCR |
| *wbpM*-RT-R | ATAGATGACCACCCTGGGCAG | RT-qPCR |
| *wzz_1_-*RT-F | TGCCTGAGCGGTATACTGTCATC | RT-qPCR |
| *wzz_1_*-RT-R | TTCGCGCTGAGCATTACCTC | RT-qPCR |
| *wzz_2_-*RT-F | AACCAGGATGCCTTCAGCATG | RT-qPCR |
| *wzz_2_*-RT-R | AGCATGCCGTTGAGAATGGC | RT-qPCR |
| *lptC-*RT-F | TGAAGCTGGAGCACCAGAAAG | RT-qPCR |
| *lptC*-RT-R | TTGCCTTTCGGTCCGACTTC | RT-qPCR |
| *lptD-*RT-F | AGTACGTGATCCACAAGGCC | RT-qPCR |
| *lptD*-RT-R | TTGACGTTGTTGCCCTTCAGG | RT-qPCR |
| *lptE-*RT-F | AGTTCGAGCTGACCAACACG | RT-qPCR |
| *lptE*-RT-R | AACCGATCAGGTTGTTTTCGTCG | RT-qPCR |
| *lptF-*RT-F | TGCTGTTGAACAAGCAGGATACCC | RT-qPCR |
| *lptF*-RT-R | TTCCTGGTTGCTGCTGTTGAG | RT-qPCR |
| *fabY*  promoter sequence | TCGGGCGAAGGGCGGATAGCGCCACGCGTCATCCGCCGCCTCCGCAAACGCCCCAGCGCCGCGCCTCTGCGCGCGGACTTTCAAGCAAGTCCCGCAACACTCGCCCACGCACCACCTCTGCCAACAGCCCGGCCATGGGCGTCAATCCCCCCCACGGCAGACCGCCAGCACTTGTGGAAAGCCCACGCAACGCGGTATTCAGTAGGAAAATACGTGACTCGATAGTCATGATTCACGTCAGCATAGTCACCCTGATCATTTCAACGGCCACTGGCTCGTGCCCAGGTCCGTGAAGCTCGCCTCGCCGACGGGAAAGGCCGCCATCGCCGCCACGCGCTTCCCGCTCCGGACCAGGCAGCAGCCCGGCACGCGAACGACTATTCGAAGGAGTTTCTGAGTG | EMSA |


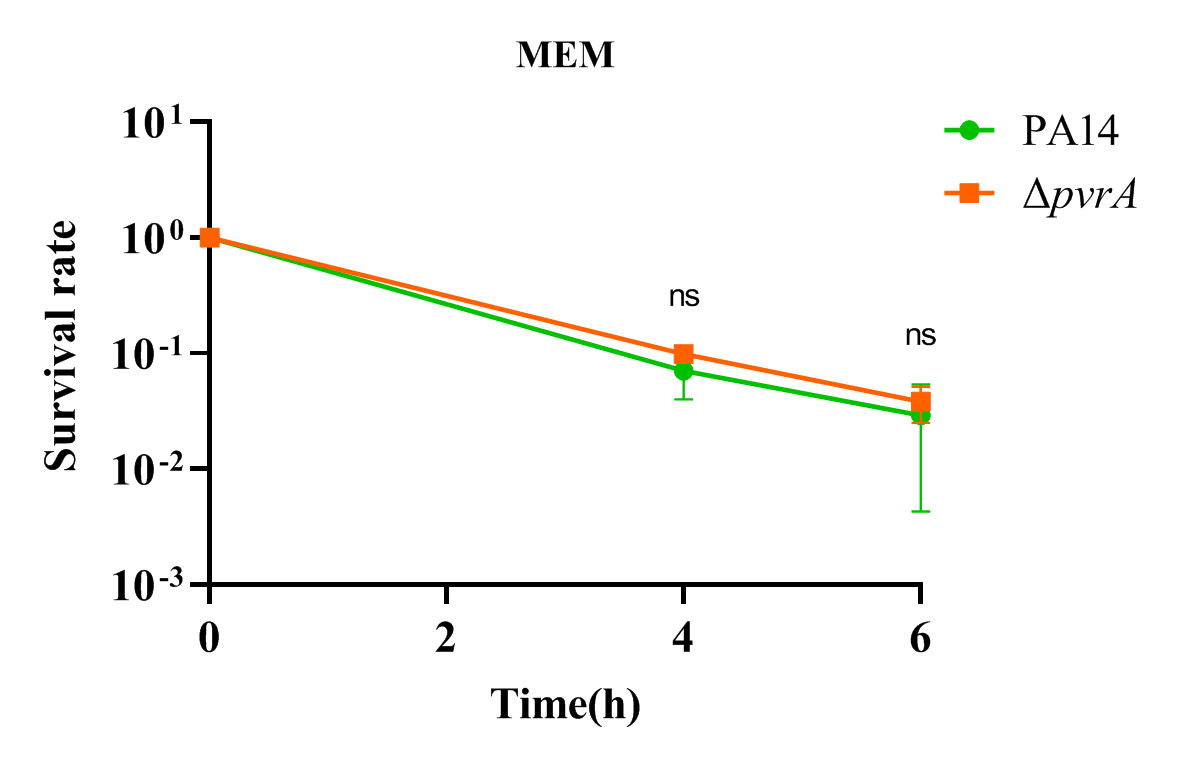

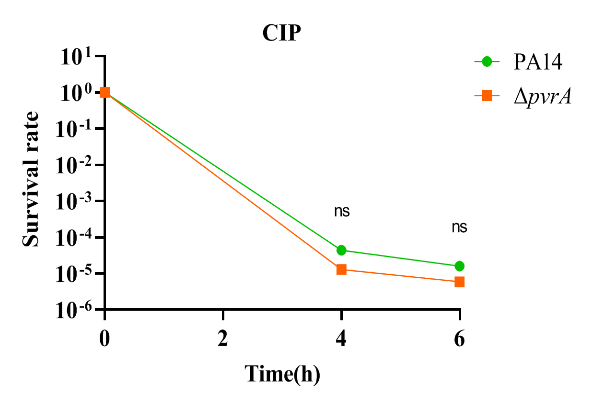


**Fig. S1** Time-kill curves of PA14 and the Δ*pvrA* mutant. Bacteria were treated with 0.5 µg/mL ciprofloxacin (CIP), 8 µg/mL meropenem (MEM) in the FA-M9 medium.


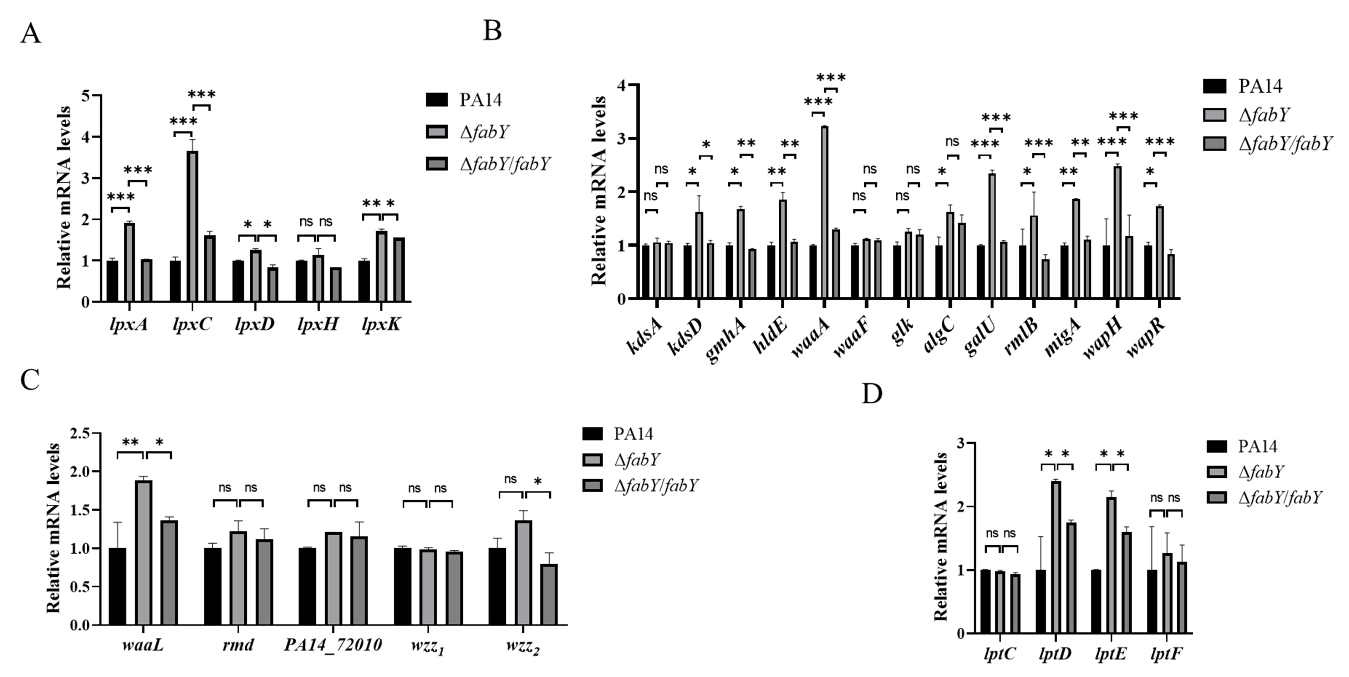


**Fig. S2** Expression levels of genes related to LPS synthesis and transportation were determined by RT-qPCR. Bacteria were grown in CA-MHB to an OD_600_ of 1.0. Expression levels of lipid A (A), core polysaccharide (B), O antigen (C) synthesis and LPS transportation and assembly related genes (D). *, P<0.05, **, P<0.01, ***, P<0.001, by Student's *t* test. The data represents the average standard deviation of the results of three samples.


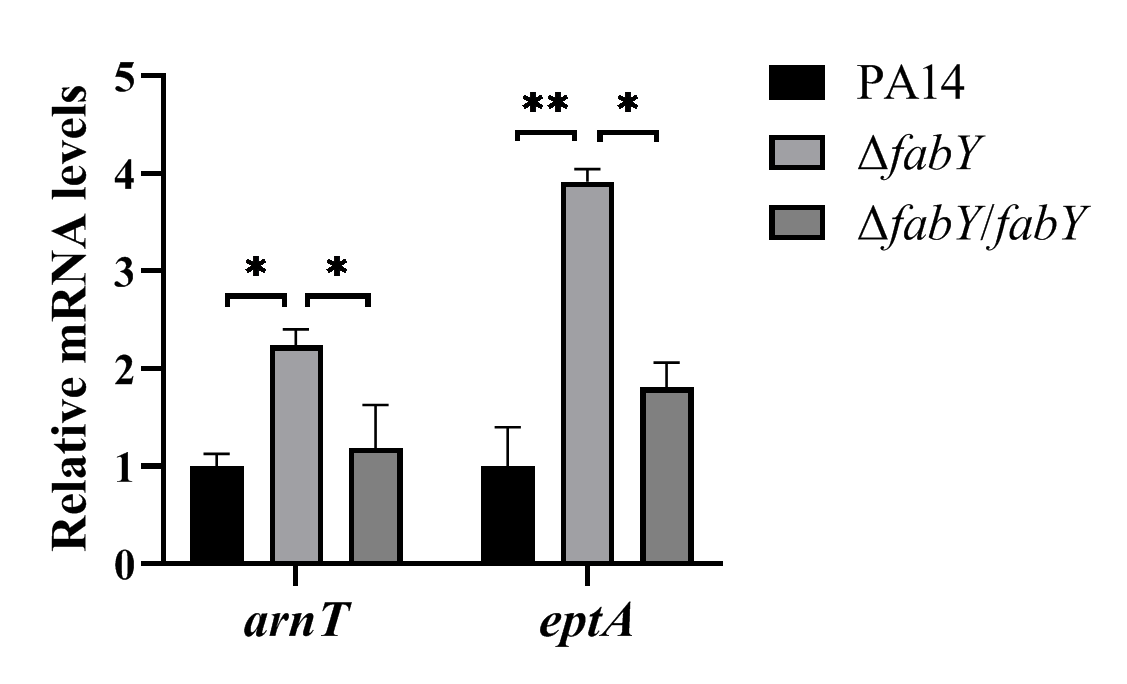


**Fig. S3** The expression levela of *arnT* and *eptA* were determined by RT-qPCR. Bacteria were grown in CA-MHB to an OD_600_ of 1.0. *, P<0.05, **, P<0.01, by Student's *t* test. The data represents the average standard deviation of the results of three samples.

**
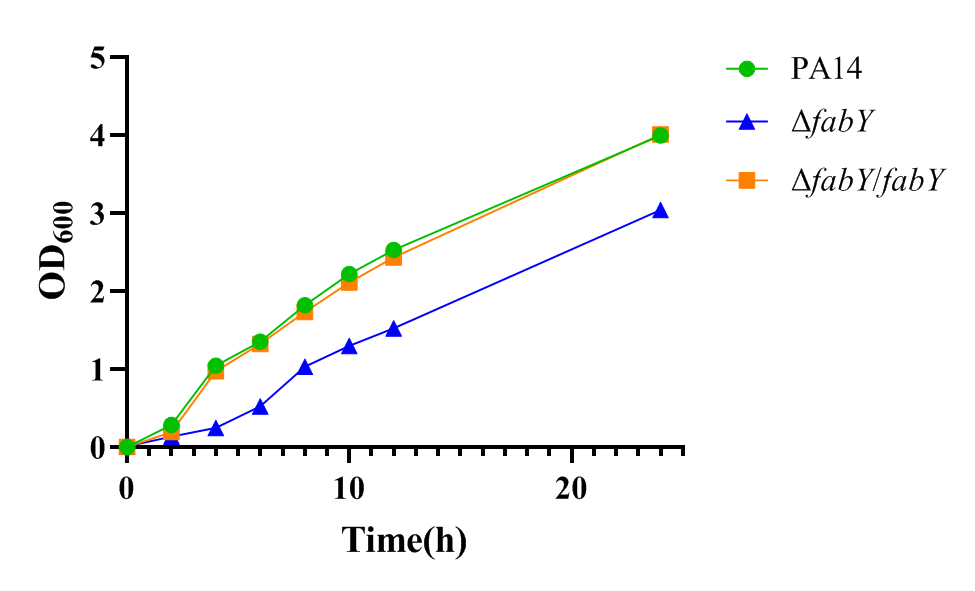
**

**Fig. S4** Bacterial growth curve. Bacteria were cultured in LB overnight for 12 hours and then transferred to CA-MHB culture medium according to the ratio of 100: 1, and the number of bacteria was measured by sampling at the specified time point.

**
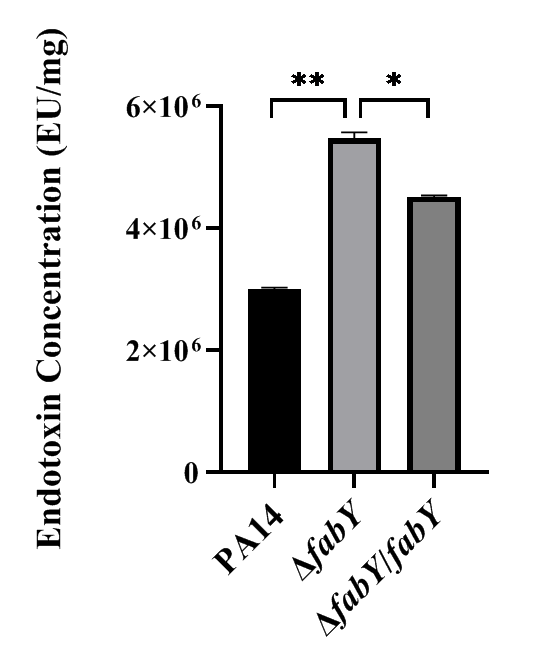
**

**Fig. S5** LPS levels were determined LAL quantification. LPS was extracted by heating at 70 °C for 15 minutes. *, P < 0.05; **, P < 0.01 by student's t test. The data represent the mean ± standard deviation of the results of three samples.

**Table. S2** MIC of *Pseudomonas aeruginosa* wild-type strain, Δ*pvrA* mutant and Δ*fabY* mutant to colistin.

| **Strain** | **MIC (μg/mL)** |
| --- | --- |
| PA14 | 1 |
| Δ*pvrA* | 1 |
| Δ*fabY* | 1 |

**Table. S3** Function and expression of genes related to LPS synthesis.

|  | Tested gene | Operon | Function | Fold change (Δ*fabY*/WT) |
| --- | --- | --- | --- | --- |
| Lipid A  synthesis | *lpxA* (*PA14_17210*) | *lpxA* (*PA14_17210*), *lpxB* (*PA14_17220*) | O-acyltransferase, responsible for the reversible acylation of UDP-D-GlcNAc in the first step of lipid A biosynthesis | 6.52±0.11 |
|  | *lpxC* (*PA14_57260*) | - | UDP-monoacyl-GlcN deacetylase | 5.09±0.32 |
|  | *lpxD* (*PA14_17180*) | - | transferr R-3- 2-N-acyltransferase, hydroxydodecanoate to the 2′ amine of UDP-3-O-(3-hydroxydecanoyl) utilizing an ACP donor | 3.15±0.48 |
|  | *lpxH* (*PA14_41400*) | - | UDP-diacyl-GlcN pyrophosphatase | 2.02±0.10 |
|  | *lpxK* (*PA14_25510*) | *lpxK* (*PA14_25510*), *kdsB* (*PA14_25530*) | Lipid A precursor disaccharide kinase | 2.20±0.21 |
| Core polysaccharide synthesis | *kdsA* (PA14_17310) |  | Kdo-8-phosphate synthase | 3.58±0.00 |
|  | *kdsD* (PA14_57890) | *kdsD* (PA14_57890) , *kdsC* (PA14_57900) | D-Arabinose 5-phosphate isomerase  ADP-L-b-D-heptose biosynthesis/ | 3.40±0.08 |
|  | *gmhA* (PA14_57500) | - | Sedoheptulose 7-phosphate isomerase | 5.38±0.64 |
|  | *hldE* (PA14_66060) | - | D-b-D-heptose 7-phosphate kinase/  D-b-D-heptose 1-phosphate adenylyltransferase | 3.18±0.55 |
|  | *waaA* (PA14_65960) | - | KDO transferase, Glycosyltransferase (GT-30) of Kdo, responsible for the initiation of the connection to lipid A | 2.94±0.07 |
|  | *waaF* (PA14_66250) | *waaF* (PA14_66250), *waaC* (PA14_66240), *wapG* (PA14_66200) | Glycosyltransferase (GT-9) of Hep^II^ | 4.91±0.59 |
|  | *glk* (PA14_22930) | - | Glucokinase | 2.43±0.12 |
|  | *algC* (PA14_70270) | - | Phosphoglucomutase/phosphomannomutase | 1.44±0.22 |
|  | *galU* (PA14_38350) | - | UDP-D-glucose pyrophosphorylase | 2.95±0.12 |
|  | *rmlB* (PA14_68170) | *rmlB* (PA14_68170), *rmlD* (PA14_68190), *rmlA* (PA14_68200), *rmlC* (PA14_68210) | dTDP-D-glucose 4,6-dehydratase | 3.15±0.25 |
|  | *migA* (PA14_55180) | - | Glycosyltransferase, responsible for Glycoform 1_(O-)_ synthesis | 3.24±0.10 |
|  | *wapH* (PA14_66160) | - | Glycosyltransferase (GT-4) possibly of Glc^II^ | 3.13±0.25 |
|  | *wapR* (PA14_66110) | - | Glycosyltransferase, responsible for Glycoform 2_(O+)_ synthesis. | 2.62±0.51 |
| O-antigen synthesis | *waaL* (PA14_66100) | - | O polysaccharide ligase | 1.84±0.09 |
|  | *rmd* (PA14_72000) | *rmd* (PA14_72000), *gmd* (PA14_71990), *wbpW* (PA14_71970), *wzm* (PA14_71960), *wzt* (PA14_71940) | GDP-D-Rha synthase | 1.59±0.18 |
|  | *PA14_72010* | PA14_72010, PA14_72020, PA14_72030, PA14_72050 | Glycosyltransferase (GT-4) | 2.59±0.07 |
|  | *wbpM* (PA14_23470 ) | - | UDP-D-GlcNAc 4,6-dehydratase | 3.80±0.20 |
|  | *wzz_1_* (PA14_23360) | - | Regulating O-specific antigen chain length which confer the production of “long” chain lengths | 3.21±0.08 |
|  | *wzz_2_* (PA14_52130) | - | confer the production of “very long” chain lengths | 2.79±0.30 |
| Transportation | *lptC (PA14_57910)* | *lptC* (PA14_57910), *lptA* (PA14_57920), *lptB* (PA14_57930) | Part of LptB₂FGC transport complex; Cooperate with LptA to transport LPS from the inner membrane to the outer membrane | 2.73±0.49 |
|  | *lptD (PA14_07770)* | - | Transfer LPS through the outer membrane and insert into the outermost leaflet of the outer membrane | 3.86±0.08 |
|  | *lptE (PA14_12210)* | - |  | 2.43±0.00 |
|  | *lptF (PA14_14500)* | *lptF* (PA14_14500) , *lptG* (PA14_14510) | Part of LptB₂FGC; Pass the extracted LPS to LptC and LptA. | 5.18±0.02 |

**References**

1. Kang, Y., *et al*., The *Pseudomonas aeruginosa* PsrA responds to long-chain fatty acid signals to regulate the fadBA5 beta-oxidation operon. Microbiology (Reading), 2008. **154**(Pt 6): p. 1584-1598.

2. Sun, Z., *et al*., PrtR homeostasis contributes to *Pseudomonas aeruginosa* pathogenesis and resistance against ciprofloxacin. Infect Immun, 2014. **82**(4): p. 1638-47.

3. Yang, B., *et al*., Identification of Novel PhoP-PhoQ Regulated Genes That Contribute to Polymyxin B Tolerance in *Pseudomonas aeruginosa*. Microorganisms, 2021. **9**(2).

4. Akhoundsadegh, N., C.R. Belanger, and R.E.W. Hancock, Outer Membrane Interaction Kinetics of New Polymyxin B Analogs in Gram-Negative Bacilli. Antimicrob Agents Chemother, 2019. **63**(10).

5. Helander, I.M. and T. Mattila-Sandholm, Fluorometric assessment of gram-negative bacterial permeabilization. J Appl Microbiol, 2000. **88**(2): p. 213-9.

6. Liberati, N.T., *et al*., An ordered, nonredundant library of *Pseudomonas aeruginosa* strain PA14 transposon insertion mutants. Proc Natl Acad Sci U S A, 2006. **103**(8): p. 2833-8.

7. Pan, X., *et al*., PvrA is a novel regulator that contributes to *Pseudomonas aeruginosa* pathogenesis by controlling bacterial utilization of long chain fatty acids. Nucleic Acids Res, 2020. **48**(11): p. 5967-5985.

8. Choi, K.H. and H.P. Schweizer, mini-Tn7 insertion in bacteria with single attTn7 sites: example *Pseudomonas aeruginosa*. Nat Protoc, 2006. **1**(1): p. 153-61.

9. Xiang, L., *et al*., CRISPR‐dCas9‐mediated knockdown of prtR, an essential gene in *Pseudomonas aeruginosa*. Letters in Applied Microbiology, 2020. **71**(4): p. 386-393.
